# Optimization of Preoperative Lymph Node Staging in Patients with Muscle-Invasive Bladder Cancer Using Radiomics on Computed Tomography

**DOI:** 10.3390/jpm12050726

**Published:** 2022-04-30

**Authors:** Martijn P. A. Starmans, Li Shen Ho, Fokko Smits, Nick Beije, Inge de Kruijff, Joep J. de Jong, Diederik M. Somford, Egbert R. Boevé, Ed te Slaa, Evelyne C. C. Cauberg, Sjoerd Klaver, Antoine G. van der Heijden, Carl J. Wijburg, Addy C. M. van de Luijtgaarden, Harm H. E. van Melick, Ella Cauffman, Peter de Vries, Rens Jacobs, Wiro J. Niessen, Jacob J. Visser, Stefan Klein, Joost L. Boormans, Astrid A. M. van der Veldt

**Affiliations:** 1 Department of Radiology and Nuclear Medicine, Erasmus MC, 3015 GD Rotterdam, The Netherlands; l.ho@erasmusmc.nl (L.S.H.); f.smits.1@erasmusmc.nl (F.S.); w.niessen@erasmusmc.nl (W.J.N.); j.j.visser@erasmusmc.nl (J.J.V.); s.klein@erasmusmc.nl (S.K.); a.vanderveldt@erasmusmc.nl (A.A.M.v.d.V.); 2 Department of Medical Oncology, Erasmus MC Cancer Institute, 3015 GD Rotterdam, The Netherlands; n.beije@erasmusmc.nl (N.B.); i.dekruijff@erasmusmc.nl (I.d.K.); 3 Department of Urology, Erasmus MC, 3015 GD Rotterdam, The Netherlands; j.j.dejong@erasmusmc.nl (J.J.d.J.); j.boormans@erasmusmc.nl (J.L.B.); 4 Department of Urology, Canisius-Wilhelmina Hospital, 6532 SZ Nijmegen, The Netherlands; r.somford@cwz.nl; 5 Department of Urology, Franciscus Gasthuis & Vlietland, 3045 PM Rotterdam, The Netherlands; e.boeve@franciscus.nl; 6 Department of Urology, Isala, 8025 AB Zwolle, The Netherlands; e.te.slaa@isala.nl (E.t.S.); e.c.c.cauberg@isala.nl (E.C.C.C.); 7 Department of Urology, Maasstad, 3079 DZ Rotterdam, The Netherlands; klavero@maasstadziekenhuis.nl; 8 Department of Urology, Radboud UMC, 6525 GA Nijmegen, The Netherlands; toine.vanderheijden@radboudumc.nl; 9 Department of Urology, Rijnstate, 6815 AD Arnhem, The Netherlands; cwijburg@rijnstate.nl; 10 Department of Oncology, Reinier de Graaf Gasthuis, 2625 AD Delft, The Netherlands; a.vandeluijtgaarden@rdgg.nl; 11 Department of Urology, St Antonius Ziekenhuis, Nieuwegein, 3543 AZ Utrecht, The Netherlands; h.van.melick@antoniusziekenhuis.nl; 12 Department of Urology, Zuyderland, 6162 BG Sittard, The Netherlands; e.cauffman@zuyderland.nl (E.C.); pe.devries@zuyderland.nl (P.d.V.); re.jacobs@zuyderland.nl (R.J.)

**Keywords:** bladder cancer, computed tomography, machine learning, radiomics

## Abstract

Approximately 25% of the patients with muscle-invasive bladder cancer (MIBC) who are clinically node negative have occult lymph node metastases at radical cystectomy (RC) and pelvic lymph node dissection. The aim of this study was to evaluate preoperative CT-based radiomics to differentiate between pN+ and pN0 disease in patients with clinical stage cT2-T4aN0-N1M0 MIBC. Patients with cT2-T4aN0-N1M0 MIBC, of whom preoperative CT scans and pathology reports were available, were included from the prospective, multicenter CirGuidance trial. After manual segmentation of the lymph nodes, 564 radiomics features were extracted. A combination of different machine-learning methods was used to develop various decision models to differentiate between patients with pN+ and pN0 disease. A total of 209 patients (159 pN0; 50 pN+) were included, with a total of 3153 segmented lymph nodes. None of the individual radiomics features showed significant differences between pN+ and pN0 disease, and none of the radiomics models performed substantially better than random guessing. Hence, CT-based radiomics does not contribute to differentiation between pN+ and pN0 disease in patients with cT2-T4aN0-N1M0 MIBC.

## 1. Introduction

At present, the recommended treatment for patients with non-metastatic muscle-invasive bladder cancer (MIBC) is neoadjuvant chemotherapy (NAC), which is followed by radical cystectomy (RC) and pelvic lymphadenectomy [1]. Approximately 50% of all patients with bladder cancer who undergo curative treatment with RC or (chemo)radiation develop distant metastases [2,3]. Cisplatin-based NAC results in a significant but relatively small overall survival benefit of 6% at ten years (hazard ratio 0.84 (95% confidence interval (CI), 0.72–0.99 *p* = 0.037) [4]. As the survival benefit of NAC is limited and the chemotherapy is associated with toxicity [5,6,7], the use of NAC in patients with MIBC is low [8,9,10]. The presence of lymph node metastases is a negative prognostic factor in patients with MIBC [11], and patients with nodal metastases at pelvic lymph node dissection, i.e., pathological N status (pN) pN+, have a 5-year survival of only 25% [12]. Hence, there is a need for an improved selection method to identify which patients could benefit from NAC in order to improve recurrence-free and overall survival.

Currently, preoperative staging of MIBC mainly relies on imaging [13,14], primarily using computed tomography (CT) of the abdomen and pelvis [15,16]. To detect malignant lymph nodes on CT, a slice thickness of preferably ≤3 mm is considered optimal [17]. Thicker slices statistically significantly lower the accuracy of LN diameter estimation on CT, regardless of LN size [18]. In clinical practice, the primary task of radiologists is to determine lymph node involvement on the CT scans, for which several characteristics have been defined in the literature. First, the short-axis diameter of lymph nodes is assessed according to the tumor–node–metastasis (TNM) guidelines developed by the American Joint Commission on Cancer [19,20]. Lymph nodes with a short-axis diameter ≥10 mm are considered malignant (N+), <10 mm are considered benign (N0) [14,21]. For response evaluation during systemic treatment (e.g., chemotherapy), lymph nodes with maximum short-axis diameter (MSAD) >15 mm on CT are used as target lesions according to response evaluation criteria in solid tumors (RECIST version 1.1) [14]. Second, the location of the primary tumor is taken into account, since the pattern of metastatic dissemination is usually associated with the location of the primary tumor [22]. In patients with MIBC, the primary lymphatic landing sites include the internal iliac, external iliac, obturator and common iliac regions up to the uretero-iliac crossing [23]. Therefore, lymph nodes along the ipsilateral lymphatic drainage should be carefully examined, even in case of minimal increase [22]. Third, the shape of lymph nodes can contribute to the differentiation of malignant from benign lymph nodes, as benign lymph nodes usually have an ovoid shape and central fatty helium [21,24]. Additional imaging characteristics of malignant lymph nodes have been identified in the literature for different imaging modalities, including short-axis-to-long-axis ratio [25], irregular border contour [26] and heterogeneity in signal intensity [26].

Prior to surgery, there is a high risk of clinical under-staging of lymph nodes in patients with bladder cancer [27,28]. In approximately 25% of patients with clinical N status (cN) cN0 MIBC, occult lymph node metastases are detected at RC and pelvic lymph node dissection [29]. Hence, to identify patients who could benefit from NAC, there is a need for a diagnostic tool to improve preoperative clinical lymph node staging in patients with MIBC.

In recent years, radiomics has been successfully used in various clinical areas [30]. In radiomics, a large number of quantitative medical imaging features and data characterization algorithms are used to predict disease characteristics or underlying pathophysiology of tissue. Regarding lymph node staging in patients with bladder cancer, Wu et al. [31] have developed a radiomics nomogram for preoperative prediction of lymph node metastases. The model was based on a 2D cross-sectional region of interest (ROI) of the primary tumor. However, bladder imaging can be affected by the position and filling of the urinary bladder, prior biopsy or inflammation, which could be misinterpreted as extravesical extension [32]. Therefore, and since the visual preoperative staging of MIBC mainly relies on lymph node characteristics, the characterization of locoregional lymph nodes may be more useful for preoperative staging.

The aim of this study was to evaluate the diagnostic accuracy of radiomics to differentiate between pN+ and pN0 disease in patients with clinical-stage (c)T2-T4a N0-N1 M0 MIBC based on preoperative CT imaging of the lymph nodes. To this end, a radiomics model was developed and validated in a post hoc analysis of the prospective CirGuidance trial [33]. In this multicenter trial, conducted between October 2013 and April 2018, 273 patients with cT2-T4a N0-N1 M0 MIBC underwent RC and were selected for NAC according to the presence of circulating tumor cells (CTCs) in the blood. In addition, the developed model was compared to the approach based on the primary tumor by Wu et al. [31].

## 2. Materials and Methods

### 2.1. Inclusion and Exclusion Criteria of the CirGuidance Trial

Inclusion criteria of the prospective CirGuidance trial (Netherlands Trial Register NL 3954) [33] were the following: (1) age ≥ 18 years; (2) histopathologically confirmed cT2-T4a N0-N1 MIBC; and (3) candidate for radical local treatment consisting of RC and pelvic lymph node dissection, as judged by the local urologist. Exclusion criteria were the following: (1) presence of metastases on CT of abdomen and chest within six weeks prior to registration; (2) presence of >50% of non-urothelial variant histology; (3) history of another malignancy within the past five years; (4) known or suspected prostate cancer; and (5) intention to treat with systemic adjuvant therapy after RC.

### 2.2. Study Design of the Post Hoc Analysis

In this study, we performed a post hoc analysis of the CirGuidance trial. The post hoc analysis was performed in accordance with the Dutch Code of Conduct for Medical Research of 2004, the Declaration of Helsinki, and it was approved by the local institutional review board (MEC-2020-0753). As the study was retrospectively performed with anonymized data, the need for informed consent was waived. For this post hoc analysis, the following inclusion criteria were applied: (1) signed informed consent for the CirGuidance trial; (2) completed treatment with RC and lymph node dissection; and (3) presence of a baseline CT urogram or contrast-enhanced CT of abdomen in either the portal venous phase or delayed phase prior to surgery. Patients were excluded if the pathology report of lymph node dissection was missing.

### 2.3. Data Collection

In the CirGuidance trial, clinical data (i.e., age and sex) and pN status were previously collected from electronic case report forms. For the current analysis, preoperative CT scans were additionally collected.

The cN was determined preoperatively by a local radiologist, and the pN was determined postoperatively by a local pathologist. The results of the postoperative pathological examination were used as reference standard for our post hoc analyses. To this end, patients were categorized based on the results of the pathological examination as pN0 (i.e., no lymph node metastases detected) or pN+ (i.e., one or more lymph node metastases detected). As no detailed description of the number or location of the dissected lymph node metastases was available, individual labeling of the lymph nodes was not possible.

For patients treated at Erasmus MC, the pathological reports were available, and details such as location and size of the malignant lymph nodes were collected. 

### 2.4. Segmentation

A medicine MSc student (L.S.H.) reviewed all preoperative CT scans slice by slice. First, all visible lymph nodes within the pelvic dissection area were manually segmented using an in-house-developed segmentation software [34]. This covers lymph nodes in eight anatomical and bilateral regions: common iliac, internal iliac, external iliac and obturator nodes. The segmentation area was proximally defined by the bifurcation of the aorta and distally by the inguinal canal in which the genitofemoral nerve is located. Segmentation was performed per slice in the 2D transverse plane, resulting in 3D volumes. Second, similar to Wu et al. [31], the largest 2D axial cross-sectional area of all visible primary tumors was segmented. 

To ensure segmentation accuracy, all segmentations were reviewed by an experienced abdominal radiologist (F.S., 7 years of experience) and manually corrected when required. Both readers were blinded for pathological N status.

### 2.5. Radiomics

**Figure 1:**
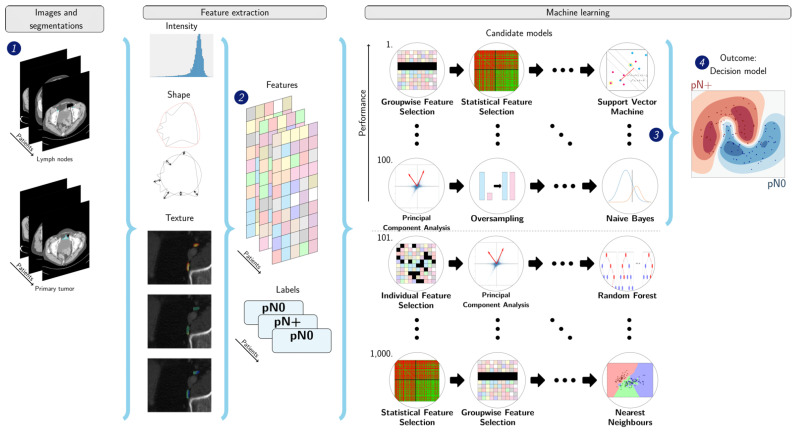
A schematic overview of the radiomics approach]. Inputs to the analyses were computed tomography (CT) of abdomen or CT urogram and corresponding segmentations of the lymph nodes (1). From these segmentations, 564 features quantifying intensity, shape and texture were extracted (2). A decision model was created (4) using the 100 best performing models (3) of 1000 candidate models. Adapted from Vos et al. [39]: the images under (1), texture features, numbers at (3), and output at (4) have been modified with respect to the original figure.

The radiomics analysis were performed using the Workflow for Optimal Radiomics Classification (WORC) toolbox [35,36]; an overview of the radiomics methodology is depicted in Figure 1. For each ROI (e.g., per lymph node or primary tumor segmentation), 564 features quantifying intensity, shape and texture were extracted from the CT scan. These include features quantifying most of the characteristics used in the visual assessment by radiologists for the preoperative staging (short-axis diameter, long-axis diameter, heterogeneity in signal intensity, border irregularity, volume and circularity). Lymph node segmentations with less than five voxels were excluded, as these volumes were considered too small for extracting a large part of the radiomics features. Additionally, to quantify the primary tumor properties, features were extracted using the segmentation of the largest 2D axial cross-sectional area of the primary tumor as ROI.

In WORC, the decision model creation consists of several steps, e.g., feature selection, resampling and machine learning. WORC performs an automated search among a variety of algorithms for each step and determines which combination maximizes the prediction performance on the training dataset using automated machine learning. WORC includes multiple resampling strategies from the imbalanced-learn toolbox [37] to overcome class imbalance (i.e., imbalance in the distribution of patients with pN+ and pN0 disease). As the CirGuidance dataset is substantially imbalanced because relatively few patients have pN+ disease, the use of various resampling strategies was turned on in WORC. The code for the feature extraction and model creation has been published as open-source software [38].

### 2.6. Experimental Setup

Six radiomics models based on the lymph nodes were created. First, two models were created including all segmented lymph nodes (Models 1a and 1b). Second, two models (Models 2a and 2b) were created including only lymph nodes with a MSAD > 15 mm according to RECIST version 1.1 [14]. Third, two models were created using a maximum of the five largest (i.e., largest MSAD) lymph nodes (Models 3a and 3b). Since larger short-axis diameters are associated with pN+, according to the TNM guidelines, we hypothesized that the inclusion of only the largest lymph nodes might better represent the characteristics of the metastatic lymph nodes. The maximum of five was chosen to exclude a large number of small nodes, but to be high enough to reduce the chance of accidentally missing malignant lymph nodes. 

As each patient had a different number of lymph nodes, two approaches for ROI definition were evaluated. For Models a, all lymph node segmentations per CT scan were combined, creating one ROI per patient, followed by the feature extraction. For Models b, all features were extracted per segmented lymph node, followed by averaging these features per patient. Both approaches result in an equal number of features per patient (*n* = 564) as input for the radiomics model.

Lastly, Model 4 was based on the primary tumor according to Wu et al. [31]. For each patient, the features extracted from the largest 2D axial cross-sectional area of the primary tumor were used. Patients were excluded from the analyses using Model 4 when the primary tumor was not visible or the diffuse bladder wall thickening interfered with defining the contour of the primary tumor. To construct a prediction model from the radiomics features extracted from the primary tumors, similar to the other models, the WORC method was used.

### 2.7. Statistics

The evaluation of each of the radiomics models was performed using a 100× random-split cross-validation [40,41]. In each iteration, the data were randomly split into 80% for training and 20% for testing in a stratified manner to assure a similar class distribution in the training and testing sets as in the complete dataset. Model optimization was performed within the training set using an internal 5× stratified random-split cross-validation, using 80% for training and 20% for validation. Hence, all optimization of the radiomics model was based solely on the training dataset to eliminate any risk of over-fitting on the test dataset. 

The performance of the radiomics models to discriminate between patients with pN+ and pN0 disease was evaluated using the area under the curve (AUC) of the receiver operating characteristic (ROC) curve, balanced classification accuracy (BCA) [42], sensitivity and specificity. To assess the performance during training, the mean and standard deviation over the 100× cross-validation iterations are reported. The performance on the test dataset was assessed to evaluate the generalization performance on unseen data, for which both the mean and 95% CI of the metrics are stated. The corrected resampled *t*-test was used to construct 95% CIs for the performance metrics, based on the results from all 100× cross-validation iterations [40]. Confidence bands for the ROC curves were constructed using fixed-width bands [43]. 

To evaluate the predictive value of the individual radiomics features and differences in clinical and image acquisition characteristics, a Mann–Whitney U-test was used in case of continuous variables and a Chi-Square test for categorical variables. Differences were considered statistically significant when *p*-value ≤ 0.05. The *p*-values were corrected for multiple testing using the Bonferroni correction (i.e., multiplying the *p*-values by the number of tests).

The statistical analyses were performed using SPSS 25.0 (Statistical Package for Social Sciences, Chicago, IL, USA) and Python 3.7.6 (the Python Software Foundation, Beaverton, OR, USA).

## 3. Results

### 3.1. Patient Characteristics

**Figure 2:**
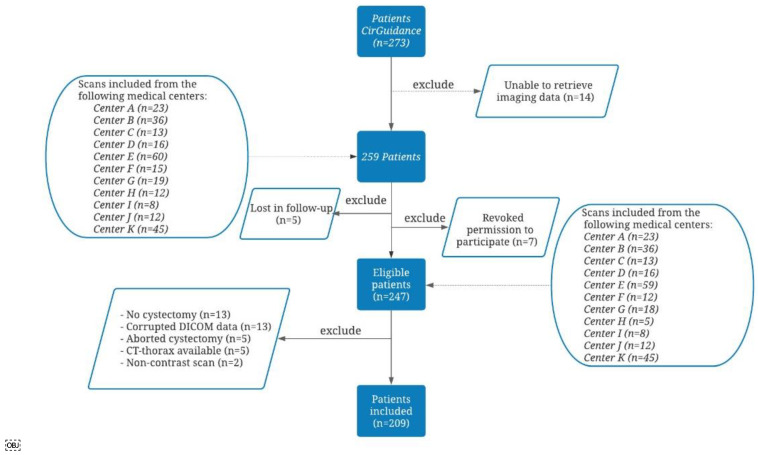
Flowchart of patients included in our post hoc analysis of the CirGuidance trial [33]. Reasons for exclusion were: lost to follow-up (*n* = 5), revoked permission to participate (*n* = 7), no cystectomy (*n* = 13), corrupted DICOM data (*n* = 13), aborted cystectomy (*n* = 5), CT-thorax provided (*n* = 5) or non-contrast scan (*n* = 2). In the end, 209 patients were included in this study.

The flowchart of the included patients is shown in Figure 2. In total, 209 patients (150 male; 59 female) with T2-T4a N0-N1M0 MIBC were included in this post-analysis. Of the fifteen medical centers that participated in the CirGuidance trial, eleven provided data from their local database.

A total of 37 patients were excluded because of the following: no cystectomy (*n* = 13), aborted cystectomy (*n* = 5), corrupted DICOM data (*n* = 13), only CT of chest available (*n* = 5) and non-contrast CT scan (*n* = 2).

Clinical and imaging characteristics of the included patients are reported in Table 1. Pathological examination showed 159 patients with pN0 disease and 50 patients with pN+ disease. Of the patients with pN+ disease, 48 patients had pN1 disease, 1 patient had pN2 disease, and 1 patient had pN3 disease. There was a statistically significant difference in sex (*p* = 0.013) but not in age (*p* = 0.639) between patients with pN+ and pN0 disease. 

Prior to surgery, 96% (48/50) of the patients with pN+ were under-staged as cN0, and 2% (3/159) of the patients with pN0 were over-staged as cN+. After surgery, 24% (48/204) of the patients with cN0 disease were up-staged, while 60% (3/5) of the patients with cN1 disease were down-staged. The median time between preoperative CT scan and surgery was 2 (range 1–9) months and did not significantly differ between patients with pN+ and pN0 disease (*p* = 0.678). The median numbers of lymph nodes removed were 18 in patients with pN+ and 16 in patients with pN0 disease; the differences were not significant (*p* = 0.106). In patients with pN+ disease, the median lymph node yield, i.e., the percentage of pathological lymph nodes of all lymph nodes removed, was 9%.

As a result of the multicentric study design of the CirGuidance trial, the dataset originated from 25 different scanners, resulting in heterogeneity in the CT acquisition protocols (Table 1). For example, the slice thickness ranged between 1.0 and 5.0 mm, the tube current between 72 and 658 mA, and the kilo voltage peak between a potential of 80 and 140. Additionally, the dataset included differences in the manufacturer (7 General Electric Medical Systems, 83 Philips, 94 Siemens, 25 Toshiba), and 22 different reconstruction kernels were used. Only peak kilovoltage (*p* = 0.040) showed statistically significant differences between patients with pN+ and pN0 disease.

**Figure 3:**
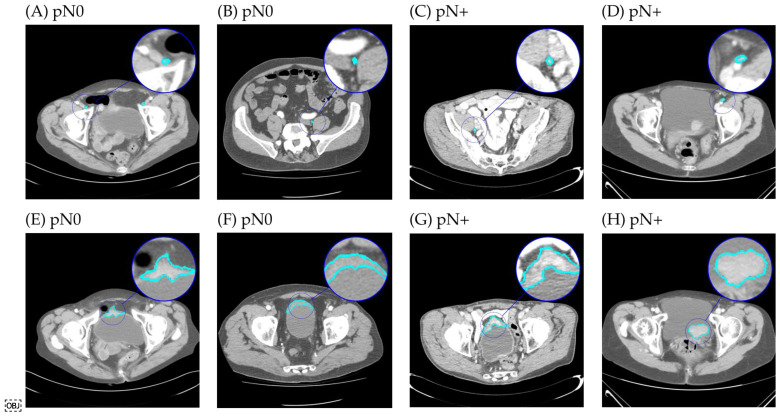
Randomly selected example segmentations on computed tomography scans of four patients with muscle-invasive bladder cancer scheduled for radical cystectomy and pelvic lymph node dissection. Top row: segmentations of pelvic lymph nodes in four patients without (pN0) (**A**,**B**) and with (pN+) (**C**,**D**) nodal metastases at pelvic dissection. Bottom row: corresponding segmentations of the largest 2D axial cross-sectional area of the primary tumor (**E**–**H**).

**Figure 4:**
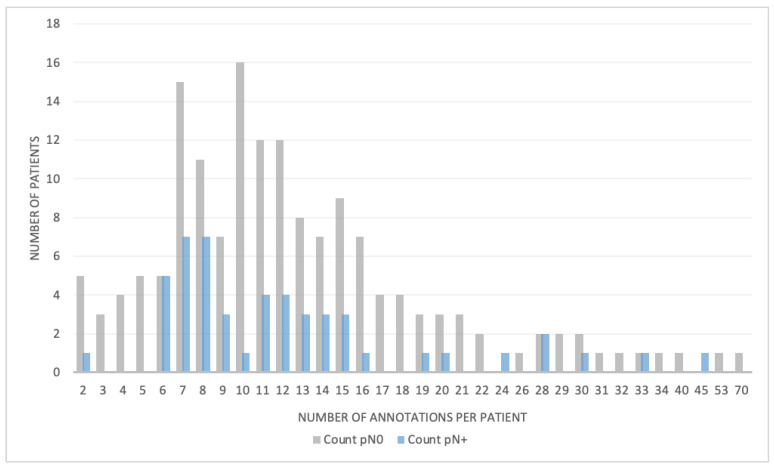
Number of segmentations per patient in patients with pN0 (gray) or pN+ (blue) muscle-invasive bladder cancer.

In total, 3153 lymph nodes were segmented. Of these, 430 segmentations were excluded, as these contained less than five voxels. Hence, 2723 lymph nodes were included in the analyses. Examples of the segmentations are depicted in Figure 3. A histogram of the distribution of lymph nodes per patient for both the pN0 and pN+ disease is shown in Figure 4. The number of lymph nodes differed substantially between patients, varying from 2 up to 70 segmentations. Between patients with pN+ and pN0 disease, there was no significant difference in the number of LN segmentations (*p* = 0.534), LN volume (*p* = 0.264) or LN size (i.e., maximum diameter) (*p* = 0.396).

In 38/209 patients (18%), the primary tumor was not visible, or the diffuse bladder wall thickening interfered with defining the contour of the primary tumor. The primary tumor could be segmented in the other 171 patients who were included in Model 4.

In the pathological examination after RC of the 31 patients included at Erasmus MC, 8 patients were classified as pN+ disease. In total, thirteen metastatic lymph nodes were found, with a median metastatic lymph node maximum diameter of 3.0mm (IQR 1.5–6.0 mm). Three patients only had lymph nodes with a maximum diameter smaller (between 0.7 and 2.5 mm) than the CT slice thickness first quartile (3.0 mm). These diameters were substantially lower than the diameters of the LN segmentations on the CT scans in patients with pN+ disease (mean ± standard deviation: 13.81 ± 9.19 mm) (see Table 1).

### 3.2. Radiomics

**Figure 5:**
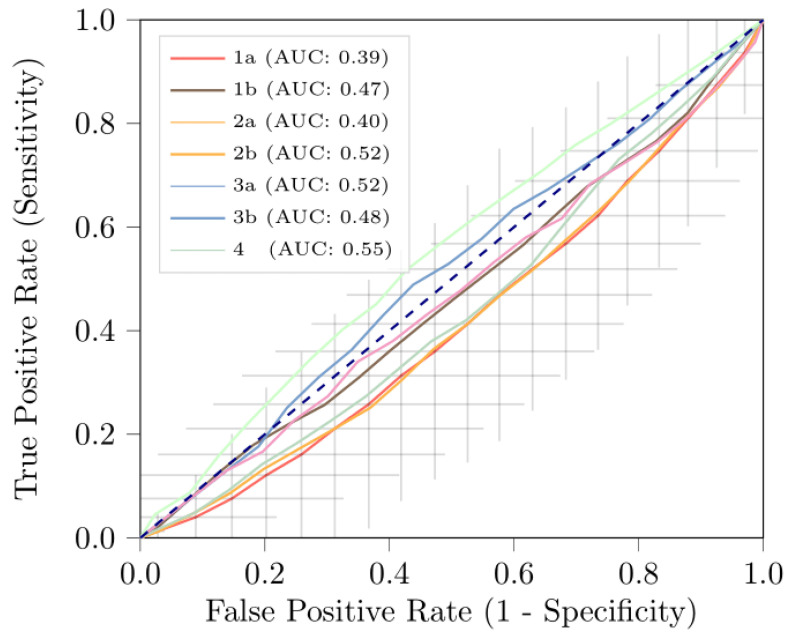
Receiver operating characteristic curves of the radiomics models in the ***testing*** datasets based on: (1a) all lymph nodes—features combined over all nodes; (1b) all lymph nodes—mean of features per lymph node; (2a) lymph nodes > 15 mm—features combined over all nodes; (2b) lymph nodes > 15 mm—features combined over all nodes; (3a) largest five lymph nodes—features combined over all nodes; (3b) largest five lymph nodes—features combined over all nodes; and (4) features extracted from the largest 2D cross-sectional area of the primary tumor. The curves represent the mean of the 100× random-split cross-validations; for Model 1a, 95% confidence intervals are represented by the crosses.

None of the radiomics features in any of the seven radiomics models showed statistically significant differences in patients with pN0 and pN+ disease. Table 2 shows the performance of the seven radiomics models for distinguishing patients with pN+ from patients with pN0 disease during testing; Figure 5 depicts the ROC curves. The performance of the models during training is depicted in Supplementary Table S1.

All models showed a good performance during training, with mean training AUCs between 0.79 and 0.92. However, during testing, all of the models based on lymph nodes had mean AUCs similar to guessing (0.50) (1a: 0.39, 1b: 0.47, 2b: 0.40, 2b: 0.52, 3a: 0.42 and 3b: 0.48). When using a cut-off value of ≥15 mm for short-axis diameter (Models 2a and 2b), a total of 403 lymph nodes were included, thereby excluding 16 patients (5 pN+; 11 pN0). When using maximally the five largest lesions (Models 3a and 3b), a total of 1017 lymph nodes were included. Model 4, which was based on features extracted from the segmentation of the largest 2D axial cross-sectional area of the primary tumor, had an AUC of 0.55. For all models, the specificity (i.e., accuracy of predicting pN0) was close to 1.00, while the sensitivity (i.e., accuracy of predicting pN+) was close to 0.00. This indicates that the models predicted most patients as pN0.

## 4. Discussion

To improve preoperative staging of patients with MIBC and the selection for NAC, the aim of our study was to evaluate CT-based radiomics for preoperative prediction of pathological lymph node status. In our study, none of the radiomics features showed significant differences between pN0 and pN+ disease, and all radiomics models performed similarly to guessing. Hence, the results show that there is no association between radiomics features of lymph nodes and the pN status in patients with MIBC. 

Although 25% of patients with cN0 MIBC are upstaged to pN+ disease [29], clinical lymph node staging of MIBC is still primarily based on preoperative CT [13,14,15,16]. Our study was designed to comprehensively evaluate the relation between CT-based radiomics features from lymph nodes and the pN status. To our knowledge, this is the first study to evaluate this relationship. 

The WORC radiomics method applied in this study has been previously validated in a variety of clinical applications [35,36]. In eleven of the twelve previous studies, the radiomics models had a better performance (mean AUCs between 0.68 and 0.94), and multiple features showed differences in univariate statistical testing, e.g., [39,44,45,46]. In the current study, none of the radiomics features had any discriminative value. Therefore, it can be concluded that radiomics features of lymph nodes cannot be applied for staging of MIBC in our dataset. WORC includes a wide variety of radiomics algorithms (e.g., machine-learning methods) and automatically optimizes the combination, thereby evaluating many different approaches. Hence, it is unlikely that other radiomics algorithms will lead to an improved performance for lymph node staging of MIBC.

During training, the models all obtained a high performance, indicating that our radiomics method is learning, and the models are not misspecified. However, in machine learning, the performance on the training dataset is generally not a good indicator of the predictive performance on unseen data due to the problem of over-fitting [40,41]. As commonly used in machine learning, we used a cross-validation to obtain an unbiased performance estimate of how the models would perform on unseen data. The performance on the test set shows poor generalization, indicating over-fitting during training. These findings support our use of cross-validation.

In our dataset, there was a statistically significant difference in sex: 76% of the pN0 patients were male, while this was only 58% in the pN+ patients. Generally, BC is more common in males than in females, with an incidence rate of approximately four times higher [47]. Additionally, in males, the percentage of BC being MIBC is higher [48]. Hence, the sex imbalance in the CirGuidance trial is in line with the literature.

The poor discriminating performance of our lymph-node-based radiomics models may be affected by two factors. First, our models were based on all visible lymph nodes, while on average, only two or three positive lymph nodes were detected at pathological examination [49,50]. As we combined the features of all visible lymph nodes per patient, the metastatic feature characteristics for the radiomics model might have been affected by the large number of benign lymph nodes that were included in our analysis. While we hypothesized that models based on only the five largest lymph nodes (Models 3a and 3b) could overcome this attenuation, these models did not yield a higher performance. Second, smaller nodes could be missed at larger CT slice thicknesses. The slice thickness of the CT scans in our study was between 3.0 and 5.0 mm, which may have resulted in missing small malignant lesions and poor visualization of medium-sized lymph nodes. This may have contributed to the poor performance of our radiomics models and may explain the substantial mismatch between clinical and pathological N status, specifically the high percentage of under-staging, as also illustrated by the substantial difference in LN size between the CT segmentations and pathological examination. However, similar poor performance has been reported at a smaller slice thickness of 1.5mm [51]. Therefore, a smaller slice thickness of CT is not considered to improve the performance of radiomics for lymph node staging in MIBC. 

In addition, we externally validated the radiomics method of Wu et al. [31], who used radiomics to predict lymph node staging in 188 patients with bladder cancer based on the largest 2D axial cross-sectional area of the primary tumor. They reported an AUC of 0.85 in the validation dataset, while our model, which was based on the same ROI and similar radiomics methods, performed similarly to guessing. This low performance could be attributed to multiple factors. First, imaging of the bladder on CT scans relies upon multiple factors, including prior biopsy, presence of inflammation and urinary filling and position of the bladder [32]. In the CirGuidance trial, no strict bladder preparation protocol is used, which may have negatively affected the applicability of the method by Wu et al. Second, Wu et al. used arterial-phase CT, which is uncommon to stage bladder cancer according to the European guidelines for urology [1]. Hence, arterial-phase CT is not by default included in the imaging protocols in The Netherlands and was thus not performed in the majority of the patients in the CirGuidance trial. Third, Wu et al. used a single-center dataset without external validation, which, as they pointed out, “limits the level of evidence”. Contrarily, we used a multicenter database with routinely acquired data representing real-world variations. These variations may have led to unwanted variations in the imaging features, which may have (negatively) impacted the performance. Using a single acquisition protocol could improve the performance unaffected by such variations, but it is not always feasible in a multicenter setting and limits the applicability. As the WORC method has previously successfully been used in similar settings [35,36], we do not expect that the poor performance can be explained by the variations in image acquisition alone.

The current study has several limitations. First, the description of pathological lymph node involvement was limited, as extensive pathological reports were not available for all patients. For this reason, during training, only a ground truth label of pN0 or pN+ disease per patient could be used. Additional specifications, e.g., location of positive lymph nodes per patient, or ideally, reference labeling per lymph node, may substantially improve the performance of radiomics models [22]. Second, as previously discussed, the high slice thickness of the CT scans in our study of 3.0–5.0 mm may have limited the reliability of LN detection and assessment, especially for small (diameter < slice thickness) lymph nodes [18]. Third, in some patients, the interval between the date of diagnosis and RC was long (up to nine months), as no specific timeframe was set between clinical lymph node staging and surgery date in the CirGuidance trial. This long interval time may have contributed to the discrepancy between cN status and pathological reports. The estimated impact on our results is, however, minimal, as there was only one patient with an interval of 9 months, and the inter-quartile range was between 2 and 3 months. Moreover, a delay of surgery may occur in daily practice [52]. Therefore, we did not consider it necessary to exclude patients with such long intervals from our analysis. Fourth, all assessments and procedures were conducted by the local clinicians and pathologists and may have led to inter-observer variability, but this is representative of daily clinical practice. As the surgery of RC and lymph node dissection could have been influenced by the experience of the surgeons and inter-operator variability [53], malignant lymph nodes may have been missed. This may have led to inaccuracies in the reference standard and thus negatively affecting the (perceived) performance of our radiomics models. 

Both our results and the literature [27,28,29] indicate poor performance of CT in detecting malignant lymph nodes in patients with MIBC. Therefore, new imaging tools are required to improve staging in these patients. Several studies have reported negative results about the addition of positron emission tomography (PET) using fluorodeoxyglucose (FDG) to CT [51,54,55,56], but its performance may be improved by the use of more disease-specific tracers in the future [57,58]. More promising results have been obtained using magnetic resonance imaging (MRI) [57]. For example, Thoeny et al. (2014) [59] showed that diffusion-weighted MRI led to higher sensitivities (0.64–0.79) in detecting lymph node metastases, with 77% of the malignant lymph nodes having maximum diameters <3 mm. In addition, irregular border contour [60] and signal intensity heterogeneity [60,61] on MRI have been shown to differentiate metastatic lymph nodes. Therefore, further research is needed to investigate the use of various MRI protocols (e.g., T2-weighted, dynamic-contrast-enhanced and diffusion-weighted MRI [62],) for the detection of lymph nodes in patients with MIBC, as well as the relatively novel PET/MRI approach [63]. Since our radiomics approach has previously been proven successful for MRI [35,36], the same approach could be evaluated to differentiate pN+ and pN0 disease based on MRI.

## 5. Conclusions

The aim of this study was to evaluate the use of radiomics to differentiate between pN+ and pN0 disease in patients with clinically node-negative MIBC based on preoperative CT scans of the pelvic lymph nodes. Our results showed that CT-based radiomics of the lymph nodes cannot discriminate between pN0 and pN+ disease. The poor performance of our radiomics models may be affected by the low sensitivity of CT in detecting malignant lymph nodes. The use of more detailed pathological information for training of the radiomics model, e.g., the location of malignant nodes or individual labeling, could improve the performance. In addition, an evaluation of other imaging modalities, specifically MRI, is needed for preoperative staging of MIBC.

## Figures and Tables

**Table 1:** Patient characteristics of the subset of 209 patients from the CirGuidance trial [33] used in the current post hoc analysis. Statistically significant *p*-values are depicted in **bold**.

	pN0 (*n* = 159)	pN+ (*n* = 50)	*p*-Value
**Sex**			**0.013**
Female N (%)	38 (24%)	21 (42%)	
Male N (%)	121 (76%)	29 (58%)	
**Age (years)** ^†^	69 [62–74]	70 [61–76]	0.639
**Clinical N status**			0.394
cN0	156	48	
cN+	3	2	
**LN segmentations, mean number per patient (±SD)**	13.13 (±8.9)	12.7 (±8.2)	0.534
**LN volume (cl), mean (±SD)**	0.030 (±0.062)	0.031 (±0.070)	0.264
**LN maximum diameter (mm), mean (±SD)**	13.81 (±9.19)	13.96 (±9.44)	0.396
**LN removed N** ^†^	16 [13–23]	18 [14–25]	0.106
Pathological LN yield (%) ^†^		9 [6–17]	
**Time between scan and surgery date (months)** ^†^	2 [2–3]	2 [2–3]	0.993
**Imaging**			
Slice thickness (mm) ^†^	5.0 [3.0–5.0]	5.0 [3.0–5.0]	0.127
Pixel spacing (mm) ^†^	0.77 [0.71–0.80]	0.75 [0.70–0.81]	0.151
Tube current (mA) ^†^	237.0 [159.0–350.0]	191.5 [147.0–318.0]	0.073
Peak kilovoltage ^†^	120 [100–120]	100 [100–120]	**0.040**

Abbreviations: SD, standard deviation; LN, lymph node; pN, pathological N status. ^†^ Values are median (inter-quartile range).

**Table 2:** Performance of the radiomics models for distinguishing pN+ from pN0 disease in patients with muscle-invasive bladder cancer in the ***testing*** datasets. Models are based on: all lymph nodes and combining segmentations per CT scan as one ROI, followed by feature extraction (1a) or extracting features from lymph nodes individually followed by averaging these features per patient (1b); lymph nodes with MSAD > 15 mm, combining segmentations per CT scan as one ROI followed by feature extraction (2a) or extracting features from lymph nodes individually followed by averaging these features per patient (2b); largest five lymph nodes, combining segmentations per CT scan as one ROI followed by feature extraction (3a) or extracting features from lymph nodes individually followed by averaging these features per patient (3b); features extracted from the primary tumor as ROI. Values are mean (95% confidence interval) over the cross-validation iterations.

	Model 1a	Model 1b	Model 2a	Model 2b
**Included LNs**	All	All	MSAD > 15 mm	MSAD > 15 mm
**Feature extraction**	All LNs as one ROI	Per LN and averaged	All LNs as one ROI	Per LN and averaged
**AUC**	0.39 [0.30, 0.48]	0.47 [0.38, 0.57]	0.40 [0.33, 0.47]	0.52 [0.42, 0.62]
**BCA**	0.50 [0.50, 0.50]	0.50 [0.50, 0.50]	0.50 [0.49, 0.50]	0.50 [0.48, 0.52]
**Sensitivity**	0.00 [0.00, 0.00]	0.00 [0.00, 0.00]	0.00 [0.00, 0.00]	0.01 [0.00, 0.04]
**Specificity**	1.00 [0.99, 1.00]	1.00 [0.99, 1.00]	1.00 [0.99, 1.00]	0.99 [0.97, 1.00]
	**Model 3a**	**Model 3b**	**Model 4**	
**Included LNs**	Largest 5 LNs	Largest 5 LNs	Primary Tumor	
**Feature extraction**	All LNs as one ROI	Per LN and averaged	Primary Tumor	
**AUC**	0.42 [0.33, 0.51]	0.48 [0.37, 0.55]	0.55 [0.46, 0.65]	
**BCA**	0.50 [0.49, 0.50]	0.50 [0.48, 0.51]	0.50 [0.46, 0.54]	
**Sensitivity**	0.00 [0.00, 0.00]	0.09 [0.00, 0.25]	0.06 [0.00, 0.15]	
**Specificity**	1.00 [0.99, 1.00]	0.92 [0.81, 1.00]	0.94 [0.87, 1.00]	

Abbreviations: LN, lymph node; AUC, area under the receiver operating characteristic curve; BCA, balanced classification accuracy; MSAD, maximum short-axis diameter; ROI, region of interest.

## Data Availability

Imaging and clinical research data are not available at this time. Programing code is openly available on Zenodo at https://doi.org/10.5281/zenodo.5558892 (accessed on 31 March 2022).

## Supplementary Materials

The following supporting information can be downloaded at: https://www.mdpi.com/article/10.3390/jpm12050726/s1, **Table S1**. Performance of the radiomics models for distinguishing pN+ from pN0 disease in patients with muscle-invasive bladder cancer in the ***training*** datasets. Models are based on: all lymph nodes and combining segmentations per CT scan as one ROI, followed by feature extraction (1a) or extracting features from lymph nodes individually followed by averaging these features per patient (1b); lymph nodes with MSAD > 15 mm, combining segmentations per CT scan as one ROI followed by feature extraction (2a) or extracting features from lymph nodes individually followed by averaging these features per patient (2b); largest five lymph nodes, combining segmentations per CT scan as one ROI followed by feature extraction (3a) or extracting features from lymph nodes individually followed by averaging these features per patient (3b); features extracted from the primary tumor as ROI. Values are mean ± standard deviation over the cross-validation iterations.
