# Supplementary material for: Optimization of Preoperative Lymph Node Staging in Patients with Muscle-Invasive Bladder Cancer Using Radiomics on Computed Tomography
Source: J Pers Med. 2022 Apr 30;12(5):726. doi: 10.3390/jpm12050726 (PMC9147130; doi:10.3390/jpm12050726)
Supplement: Supplementary file 1 [file jpm-12-00726-s001.zip › jpm-1685532-supplementary.pdf]

Supplementary Table S1. Performance of the radiomics models for distinguishing pN+ from pN0 disease in patients with muscle-invasive bladder cancer in the ***training*** datasets. Models are based on: all lymph nodes and combining segmentations per CT scan as one ROI, followed by feature extraction (1a) or extracting features from lymph nodes individually followed by averaging these features per patient (1b); lymph nodes with MSAD >15mm, combining segmentations per CT scan as one ROI followed by feature extraction (2a) or extracting features from lymph nodes individually followed by averaging these features per patient (2b); largest five lymph nodes, combining segmentations per CT scan as one ROI followed by feature extraction (3a) or extracting features from lymph nodes individually followed by averaging these features per patient (3b); features extracted from the primary tumor as ROI. Values are mean ± standard deviation over the cross-validation iterations.

**Training**	**Model 1a**	**Model 1b**	**Model 2a**	**Model 2b**
**Included LNs**	All	All	MSAD > 15 mm	MSAD > 15 mm
**Feature extraction**	All LNs as one ROI	Per LN and averaged	All LNs as one ROI	Per LN and averaged
**AUC**	0.88 ± 0.22	0.85 ± 0.18	0.90 ± 0.20	0.92 ± 0.15
**BCA**	0.86 ± 0.17	0.77 ± 0.20	0.89 ± 0.15	0.85 ± 0.18
**Sensitivity**	0.76 ± 0.33	0.60 ± 0.37	0.83 ± 0.27	0.74 ± 0.35
**Specificity**	0.97 ± 0.07	0.93 ± 0.19	0.96 ± 0.10	0.96 ± 0.07
**Training**	**Model 3a**	**Model 3b**	**Model 4**	
**Included LNs**	Largest 5 LNs	Largest 5 LNs	Primary Tumor	
**Feature extraction**	All LNs as one ROI	Per LN and averaged	Primary Tumor	
**AUC**	0.80 ± 0.23	0.91 ± 0.19	0.79 ± 0.20	
**BCA**	0.77 ± 0.20	0.86 ± 0.17	0.78 ± 0.17	
**Sensitivity**	0.59 ± 0.37	0.81 ± 0.29	0.68 ± 0.29	
**Specificity**	0.95 ± 0.09	0.92 ± 0.17	0.88 ± 0.17	

Abbreviations: LN, lymph node; AUC, area under the receiver operating characteristic curve; BCA, balanced classification accuracy; MSAD, maximum short-axis diameter; ROI, region of interest.
